# The East and West

**Published:** 1838

**Authors:** 


ART. II.—THE EAST AND WEST.

In the new, spirited and interesting Semi-monthly sheet (already favorably noticed by us) under the title of the Philadelphia "MEDICAL EXAMINER," we find (June 6) the following Editorial:

"DR. DRAKE, of the Cincinnati Medical College, announces that he is preparing for the press a work on the elements of Pathology. It is intended, he informs us, chiefly for circulation in the valley of the Mississippi, "as he does not anticipate for it a favorable reception East of the mountains!" Does the Doctor, then, set no value on tramontane medical criticism! Can he hold no communion with any of the varieties of opinion that are current here? Or does he suppose, that, from sectional jealousy, his Atlantic brethren are unwilling to do justice to the merits of the West? We cannot impute to Dr. Drake a design to foist his writings into popularity, by fomenting provincial prejudices; we know and appreciate his devotion to the interests of his locality; but we did not expect from him the unphilosophic illiberality, that is evinced in the attempt, (if we are right in giving this construction to his language,) to isolate the valley of the Mississippi from the general republic of medical literature. We are especially adverse to such an isolation, in the case of an author, like Dr. Drake, from the productions of whose talents, the East and West, North and South, must assert their equal claims for edification."

We feel as little conscious of meriting the respectful compliment with which this article closes, as of being obnoxious to the censure cast upon us in its preceding paragraphs. The charge (made by imputation) of "fomenting provincial prejudices," is one to which we plead not guilty. In saying that we did not 'anticipate,' for the proposed work, 'a favorable reception East of the mountains,' we simply expressed, what every observing and reflecting man would have uttered. Suppose that professor Jackson, in announcing his learned and elaborate work on the Institutes of Medicine, had said, that he should depend on America for its consumption, and did not anticipate for it 'a favorable reception' in Europe, would the observation have been thought *outre*, by any who know, that we intuitively look up, and not down the stream, for new and fresh and sparkling waters? The westward progress of the arts and sciences, is a fact about which there can be as little difference of opinion, as in relation to the effect of that progression, on our association of ideas. It requires, indeed, an effort of the mind to rise above these associations, and look into a region where intellectual developements, are of recent date, for the fruits of an ancient cultivation.

On this point we can add experience to theory. In the year 1831, we printed a limited addition of a small work, entitled "Essays on Medical Education and the Medical profession in the United States," which was well received in the West; but of a consignment made to a respectable medical Bookseller, in Philadelphia, not a single copy was sold, till they were sent to auction; nor was the work, as far as we know, noticed by any of the Journals of the East. We doubt, indeed, whether the Editors of the Examiner have ever seen it, and shall, therefore, send them a copy, as evidence that we *do* set a value on 'tramontane criticism.' Again, the present is the 45th No. of the Western Journal, commenced in the spring of 1827. A periodical to have sustained itself through 11 years, must have had patronage. But has that been Eastern, or Western, chiefly? Let our Register speak. Richmond and Washington are *not* on it, Baltimore has *one* subscriber, Philadelphia *none*, New York *one*, New Haven *one*, Providence *none*, Boston *one*. Thus eight cities of the Seaboard, the ancient seats of Medical learning, embracing nearly half a million inhabitants, and dignified with medical practitioners, who would do honor to any age or nation, have in them but four subscribers to our backwoods' quarterly. '*E Sylvis Nuncius*,' has not, indeed, procured that support, from our elder and more favored brethren, of the "old settlements," which the Examiner assures us they are ready and willing to afford. We might grant, that this want of eastern patronage may be ascribed to want of merit in our Journal, if it had, at any time, been more liberal than at present, but it has not. We must confess, however, that its merits are not very great.

But a word on the proposed elements of pathology. It has been undertaken, without any deep conviction of our qualifications for its able execution; but in compliance with the formal solicitations of two of the three medical classes of the young Cincinnati College; and under the impression, that a compendious work on that subject, not loaded with references, and of indegenous origin, would reach, and be acceptable to, the detached village and country physicians of the West. For them, therefore, it is primarily intended, while at the same time we should feel gratified with eastern patronage and endeavor to profit by eastern criticism. In the annunciation of it which we made, in the last number of the Journal, it was stated, that if a publisher could not be obtained, it would not be put to press until a sufficient number of subscribers' names were sent to us, to justify the printing of it as a subscription book. That number has not yet been received, and consequently it has not been committed to the press.

